# Barriers and facilitators in the implementation of an evidence-based health promotion intervention in a primary care setting: a qualitative study

**DOI:** 10.1108/JHOM-12-2020-0512

**Published:** 2021-09-02

**Authors:** Heather L. Rogers, Susana Pablo Hernando, Silvia Núñez - Fernández, Alvaro Sanchez, Carlos Martos, Maribel Moreno, Gonzalo Grandes

**Affiliations:** Psychology and Health Research Group, Biocruces Bizkaia Health Research Institute , Barakaldo, Spain; Ikerbasque Basque Foundation for Science , Bilbao, Spain; Primary Care Research Unit of Bizkaia, Biocruces Bizkaia Health Research Institute , Basque Healthcare Service–Osakidetza , Barakaldo, Spain; Arrigorriaga Health Center, Integrated Health Organization Barrualde-Galdakao , Osakidetza, Arrigorriaga, Spain; Integrated Health Organization Barrualde-Galdakao , Osakidetza, Galdakao, Spain

**Keywords:** Implementation science, Primary care, Health promotion, Healthcare management, Healthcare organization

## Abstract

**Purpose:**

This study aims to elucidate the health care organization, management and policy barriers and facilitators associated with implementation of an evidence-based health promotion intervention in primary care centers in the Basque Country, Spain.

**Design/methodology/approach:**

Seven focus groups were conducted with 49 health professionals from six primary care centers participating in the Prescribing Healthy Life program. Text was analyzed using the Consolidated Framework for Implementation Research (CFIR) focusing on those constructs related to health care organization, management and policy.

**Findings:**

The health promotion intervention was found to be compatible with the values of primary care professionals. However, professionals at all centers reported barriers to implementation related to: (1) external policy and incentives, (2) compatibility with existing workflow and (3) available resources to carry out the program. Specific barriers in these areas related to lack of financial and political support, consultation time constraints and difficulty managing competing day-to-day demands. Other barriers and facilitators were related to the constructs networks and communication, culture, relative priority and leadership engagement. A set of six specific barrier-facilitator pairs emerged.

**Originality/value:**

Implementation science and, specifically, the CFIR constructs were used as a guide. Barriers and facilitators related to the implementation of a health promotion program in primary care were identified. Healthcare managers and policy makers can modify these factors to foster a more propitious implementation environment. These factors should be appropriately monitored, both in pre-implementation phases and during the implementation process, in order to ensure effective integration of health promotion into the primary care setting.

## Introduction

There are challenges in translating evidence-based interventions into practice ( Morris *et al.* , 2011 ). These deficits are not knowledge gaps but issues with translation – research must be translated into practical routines and policies and scaled up appropriately ( Martin *et al.* , 2019 ). Implementation science is defined as “The scientific study of methods to promote the systematic uptake of research findings and other evidence-based practices into routine practice, and, hence, to improve the quality and effectiveness of health services and care” ( Eccles and Mittman, 2006 ). Clinical research addresses the “what”, and implementation research addresses the “how”. Implementation science methodologies serve numerous functions. They may describe or guide the research-to-practice translation process, explain what influences implementation outcomes and/or evaluate implementation ( Nilsen, 2015 ). Health care organization, management and policy factors play important roles in the successful implementation of evidence-based interventions into routine clinical care ( Fernandez *et al.* , 2018 ). This paper explores the health care organization, management and policy barriers and facilitators related to implementation of a health promotion intervention into primary care.

### Health promotion

It is well-known that preventive health care is equally important as curative health care in decreasing population mortality and morbidity ( Wang, 2018 ). As life expectancy increase and the burden of chronic disease and multi-morbidity grows ( OECD, 2020 ), one strategy to decrease the burden of chronic disease requires addressing the major risk factors, including sedentary lifestyle, smoking, unhealthy diet and alcohol misuse ( Palmer *et al.* , 2018 ). Investment in health promotion can reduce long-term health care demand and expenditures ( Domagała and Golinowska, 2020 ), and the World Health Organization (WHO) recommends that at least 3% of national health care budgets be dedicated to health promotion and disease prevention ( World Health Organization, 2018 ). The need for health care organization, management and policy changes to align with health promotion activities within the health and social care system is well-documented (e.g. Conill *et al.* , 2018 ).

Evidence of the effectiveness of health promotion on health systems performance, outcomes and sustainability is well-established ( Busse *et al.* , 2010 ). Health promotion interventions have been shown to increase healthy lifestyle behaviors, leading to chronic disease prevention and control ( Bauer *et al.* , 2014 ), and specific health promotion interventions have been found to be cost-effective in the long term ( Edwards *et al.* , 2013 ; Verhaeghe *et al.* , 2014 ). Within a health system, primary care is often designated as the appropriate setting to interface with public health and engage in health promotion activities ( World Health Organization, 2018 ). Yet there are numerous barriers that interfere with such implementation.

### Barriers and facilitators of health promotion implementation and the value of implementation science

Wändell *et al.* (2018) conducted a systematic review of barriers and facilitators in the prevention of cardiometabolic disease among health professionals in primary care. After reviewing 28 studies, these factors were grouped into five categories: structural, organizational, professional, patient-related and attitudinal. The structural barriers included time restraints and ineffective implementation of the intervention, while facilitators included adequate assistance and support and identification of challenges. The organizational barriers included lack of IT support and communication problems, whereas facilitators included structured practice of the intervention and logistical support. The professional barriers included lack of skill, knowledge and experience, with the facilitators being training and ability to motivate patients to carry out the intervention. Patient-related barriers included low adherence, with the physician-patient relationship as a facilitator. Lastly, attitudinal barriers involved a negative attitude toward prevention, with the facilitator being a positive attitude ( Wändell *et al.* , 2018 ). Clearly, contextual factors surrounding health promotion intervention implementation influence successful integration of the intervention into routine practice ( Ingram *et al.* , 2019 ).

Implementation science methodologies offer study designs, theories, models and evaluation frameworks that provide an *a priori* structure to assess and monitor key contextual factors so that differences in intervention uptake can be explained. Implementation science study designs examine the intervention separately from the implementation strategies used to achieve integration of the intervention in routine clinical practice. Thus, many theories examine dissemination of the intervention, and many models and evaluation frameworks distinguish between the clinical intervention and its implementation. The Consolidated Framework for Implementation Research (CFIR; Damschroder *et al.* , 2009 ) is a comprehensive compilation of constructs, or areas, within five separate domains. The five domains encompass: the *intervention* and any setting-specific adaptations required, the *outer setting* where the intervention is carried out (e.g. health system), the *inner setting* (e.g. a health center or the community), the *characteristics of the individuals* involved (e.g. different professionals implementing the intervention) and the *implementation process* . A systematic review has shown that the CFIR has been used across a wide range of studies ( Kirk *et al.* , 2016 ) with numerous advantages. It is well-operationalized ( Fernandez *et al.* , 2018 ) with a website ( https://cfirguide.org/ ) that contains supporting qualitative and quantitative materials. In addition, it has a meta-theoretical basis valuable for health service delivery, offering both breadth and depth of data adequate for capturing the complexity of physician-patient interventions ( Varsi *et al.* , 2015 ). In addition to using the CFIR for program evaluation and guiding stages of future implementation, the CFIR is also commonly used in program planning. The CFIR presents a useful framework to identify potential barriers and facilitators to implementation. The CFIR may also be a useful framework for conceptualize how to design or select implementation strategies to that address identified barriers and facilitators. The Expert Recommendations for Implementing Change (ERIC) represent a list of the most commonly used implementation strategies. The Eric strategies list was compiled through a modified Delphi process including 169 expert implementation scientists and practitioners ( Perry *et al.* , 2019 ; Powell *et al.* , 2015 ; Waltz *et al.* , 2015 ). The experts were asked to identify the Eric strategies that best align with CFIR-based contextual barriers (see Waltz *et al.* , 2019 ). A CFIR-ERIC matching tool developed based on the results of that study is available online ( https://cfirguide.org/choosing-strategies/ ).

Prior research with the CFIR suggests that the inner setting domain is of particular importance to distinguish among successful implementation of evidence-based interventions (e.g. Varsi *et al.* , 2015 ). The inner setting domain is especially related to health care organization, management and policy. Crable *et al.* (2020) interviewed leaders of inpatient departments at safety net hospitals serving under-served populations to understand the factors influencing the implementation of improvement initiatives. The CFIR was used to structure the interview and analyze the data. Important barriers and facilitators were identified with respect to inner setting constructs. Specific barriers were related to the “available resources” construct regarding limited staffing. Additional barriers were related to the “organizational incentives and rewards” construct since stakeholder were frustrated that the hospital did not reinvest initiative-related savings (e.g. funds related to achieving quality benchmarks) to sustain and scale-up successful initiatives. A specific facilitator was related to the “access to knowledge and information” construct as information about initiatives communicated via diverse active and passive approaches lead to successful implementation ( Crable *et al.* , 2020 ).

### Prescribing Healthy Life PVS program

The Primary Care Research Unit of Bizkaia (Osakidetza-Basque Health Service) has been using implementation science methodologies, including the CFIR, as part of a larger program of implementation research. The group has a long trajectory of examining the effectiveness of evidence-based health promotion interventions in primary care and associated implementation strategies to facilitate sustained use of these interventions. The “Prescribe Vida Saludable” (PVS) program translates into Prescribing Healthy Life. The PVS program is a complex intervention drawn from evidence-based theoretical models and intervention strategies for health behavior modification such as the social learning and planned behavior theories and the 5 A's (ask, advise, agree, assist and arrange follow-up) intervention framework ( Goldstein *et al.* , 1998 ; Whitlock *et al.* , 2002 ). The PVS program intervention is led by the professionals at local primary care centers with the involvement of the surrounding community to achieve the effective integration of healthy lifestyle promotion targeting multiple risk factors into the day-to-day primary care setting. The target behaviors of interest are smoking cessation, exercise and healthy diet.

In addition to the intervention, implementation strategies are also directly studied as part of the PVS program. As per the Medical Research Council's evaluation framework ( Craig *et al.* , 2008 ; Moore *et al.* , 2015 ), a modeling phase was carried out in four primary health care centers. A collaborative and facilitated implementation strategy was piloted, and process indicators were examined ( Grandes *et al.* , 2017b ; Sanchez *et al.* , 2009 ). In the phase II quasi-experimental pilot trial, 10% of patients visiting PVS health care centers received a prescription for lifestyle change ( Sanchez *et al.* , 2017 ). Focus groups with participants in the pilot trial analyzed using the CFIR found 11 constructs associated with successful implementation ( Martinez *et al.* , 2017 ). Only 2 of the 11 factors related to implementation were associated with health care organization: the inner setting constructs “tension for change” and “learning climate”. A number of additional CFIR constructs emerged from the discussion but were not associated with uptake of the intervention across centers. These health care organizational, management and policy factors were “external policy and incentives”, “structural characteristics”, “available resources” and “formally appointed implementation leaders”.

### Aim of the study

The aim of the present study is to elucidate the health care organization, management and policy barriers and facilitators associated with the PVS program, an evidence-based health promotion intervention and associated implementation strategies carried out in primary care centers in the Basque Country, Spain.

## Methods

### Design

This qualitative study is part of a larger hybrid effectiveness-implementation type III research study funded by the Spanish Ministry of Health and the Basque Government to determine differences in implementation effectiveness of an evidence-based health promotion program in primary care centers in the Basque region and the subsequent impact on population health. Seven primary health care centers participated between May 2016 and January 2018. Two of these centers were in Bilbao and were responsible for a population of about 8,000 individuals. One center was on the outskirts of Bilbao, connected by metro, and responsible for a population of 5,300 individuals. Three centers were located in towns outside of Bilbao – one in a larger city responsible for 12,000 individuals and two in smaller towns responsible for approximately 7,000 individuals. This qualitative sub-study, in particular, examines reasons for differences in implementation effectiveness across centers. Earlier studies examining implementation of PVS by the research group ( Martinez *et al.* , 2017 ) used the CFIR ( Damschroder *et al.* , 2009 ) and found it valuable to proceed to the next phase of research. Thus, the CFIR was used to structure the focus group facilitator's guide. Six of the seven centers were willing to participate in this study to evaluate intervention implementation and provide recommendations for improvement. These focus groups occurred approximately three months after the end of data collection for the larger study.

### Participants

Participants were recruited using previously established contacts at each primary care center. These contacts included the primary care physician (PCP) leader who performs some administrative duties and the PVS program champion at each center. The PVS program champion is an individual selected by those participating in the PVS program at a given center who facilitates PVS program implementation and is not the PCP leader. Given that part of the focus group involved evaluation of the role of the PVS program champions, they were not eligible to participate in the focus groups. Two focus groups were offered at one center in order to permit the participation of all interested professionals.

### Data collection

The seven focus groups took place in designated meeting rooms at each participating primary care center in order to maximize participation of the professionals involved. Almost all occurred at lunch time, when the morning shift was concluding and the afternoon shift was beginning. The focus groups involved between 5 and 11 participants and were carried out in March and April of 2018. Each focus group lasted between 90–120 min. Two researchers carried out the focus group interviews. SPH moderated all seven focus groups using a facilitator's guide based on the CFIR ( Damschroder *et al.* , 2009 ; https://cfirguide.org/ ). The CFIR consists of five domains that were explored in detail in each group: intervention, outer setting (e.g. community and health system), inner setting (center), characteristics of the individuals involved and the implementation process. Constructs within each domain were allowed to emerge and were not explicitly part of the facilitator's guide. HLR was the observer for each focus group, taking detailed notes of the session and recording non-verbal behaviors. Each focus group was audio-recorded and transcribed verbatim. Post-session notes were compiled by the two researchers (SPH and HLR) involved in data collection.

### Data analysis

The transcriptions were coded using the CFIR constructs within each of the five domains according to the qualitative codebook with operational definitions and inclusion and exclusion criteria for each mutually exclusive construct available at https://cfirguide.org/constructs/ . This paper's aim focuses on the barriers and facilitators a primary care-based health promotion program specific to healthcare health care organization, management and policy. These areas were mapped to CFIR constructs the CFIR constructs in Table 1 . The III. Inner Setting construct D. Implementation Change, Compatibility was divided into two sub-categories – Compatibility with Workflow and Compatibility with Values – in order to appropriately group the relevant compatibility themes that emerged from the focus group discussions.

To reduce potential bias, line-by-line coding of the transcriptions was performed by two trained coders (HLR and SPH). Coding was conducted using Atlas.ti version 7 (Atlas.ti Scientific Software Development GmbH). The initial codebook of CFIR constructs within the five domains available at https://cfirguide.org/constructs/ was used. Operational definitions and inclusion/exclusion criteria in the codebook were applied. Quotations could be assigned more than one construct. Once one transcript was coded by each coder, who was blinded to the other's work, consensus meetings were held to discuss each quotation and construct coded, refining the definitions and inclusion/exclusion criteria as necessary. The final coding of the transcript was reached by consensus and consultation of a third coder (GG) as necessary. Then the next transcript was coded by each coder and the process was repeated. Textual quotations were exported into Excel by construct, and each quotation was given a numerical rating by two coders (HLR and SNF) according to instructions available here: https://cfirguide.org/evaluation-design/qualitative-data/ . In brief, the degree to which the construct was associated with successful implementation was assigned a valence from – 2 to +2. This valence determined if the construct identified for the quotation was a barrier that impeded implementation (negative valence) or facilitator (positive valence) because it was related to successful implementation. This coding work occurred using the same process. Each coder independently coded all the quotations in a given construct, consensus meetings were held to discuss the results and once consensus had been achieved on all quotations in a construct, the coders moved on to code the set of quotations in the next construct. Quotations were reviewed by a third coder (AS) for accuracy.

Results were aggregated by construct with the proportion of barriers and facilitators within each construct determined. Quotations were selected that best illustrated the main barriers and facilitators reported. The most frequently reported barriers were ranked ordered, and then constructs were consulted to identify corresponding facilitators. In most cases, barrier-facilitator pairs were obtained within the construct. If such pairing was not available, facilitators from other constructs were consulted for appropriateness. Final mapping results were reviewed by the research team for accuracy until consensus was achieved.

### Ethical consideration

This study received ethics committee approval from the Basque Government Ethics Committee (CEIC PI2019117). All participants provided signed informed consent at the beginning of each focus group session and could opt to leave the study at any time.

## Results

### Participants

A total of 49 health center professionals participated in the seven focus groups carried out in the study – 24 primary care physicians (including pediatricians), 18 nurses (including midwives) and seven administrative assistants. Table 2 shows the breakout of participants by role by center.

Overall, participants commonly reported facilitators in the inner setting domain specifically related to D. Implementation Climate with respect to 2. Compatibility (ii) with Values and 1. Tension for Change. They commonly reported barriers in the outer setting domain specific to D. External Policy and Incentives, and in the inner setting domain related to D. Implementation Climate, 2. Compatibility (i) with Workflow, and E. Readiness for Implementation, 2. Available Resources.

More facilitators than barriers were reported in the inner setting domain related to D. Implementation Climate, 6. Learning Climate and 4. Organizational Incentives and Rewards, and E. Readiness for Implementation, 1. Leadership Engagement and 3. Access to Knowledge and Information. More facilitators than barriers were also reported in the process domain related to B. Engaging, 2. Formally Appointed Internal Implementation Leaders. In contrast, more barriers than facilitators were reported in D. Implementation Climate (General). Both barriers and facilitators were reported in the inner setting domain related to A. Structural Characteristics, B. Networks and Communications, C. Culture and D. Implementation Climate, 3. Relative priority.

Barriers and facilitators in the inner setting domain related to D. Implementation Climate, 5. Goals and Feedback and E. Readiness for Implementation (General) did not emerge from the analysis.

### III. Inner setting – D. Implementation climate, 2. Compatibility (ii) with Values

The most important facilitators of PVS program implementation fell under this construct, evaluated positively by all participants in all centers. Only facilitators were reported. The most commonly endorsed facilitators included the perception that health promotion is fundamental to a health system and specifically an important part of primary care.

A participant from center C7 described the PVS program intervention and health promotion as the most important activity in primary care:

This [the PVS program intervention] forms part of our work, part of our foundation and our reason for being. It is offering [our patients good] habits and offering them health and everyone knows that this is much more important than taking their blood pressure and prescribing a drug.

Another center C4 participant highlighted the health promotion aspect of the PVS program intervention as fundamental to primary care services:

Healthy life is essential. The [PVS program intervention] tool is not something outside of everyday practice. It is something that you believe will help you in your consultations with patients. And patients also have to know this. This [PVS program] becomes well-established, we're on the right track. This makes it easier when it comes time to implement, when it comes time to move [the PVS program] forward. It [the PVS program] is something that you can use in your consultations with patients, whether you are physician or nurse. You know what you have to do, you follow the tool's guidance. It takes time, of course, but it is part of your fundamental prescription [of healthy life].

No barriers were identified by participants in this area. The facilitators noted under this construct were related to the construct III. Inner Setting – D. Implementation Climate, 1. Tension for Change, defined as an identified need to implement these values, which emerged in only two centers. Only facilitators were reported.

### II. Outer setting – D. External policy and incentives

The most important barriers to PVS program implementation fell under this construct, evaluated negatively by all participants in all centers. All only barriers were reported. The most commonly identified barriers were: lack of support (both financial and political) to primary care, lack of specific logistical facilitators (e.g. authorizations to be away from their consultation for program-related activities and providing substitute personnel when leave is needed) and a general lack of priority given to health promotion activities by higher management (e.g. appropriate measurement metrics, ability to schedule longer appointments if needed).

A participant in center C3 stated the importance of financial investment in primary care:

It's important to invest money in primary care for many things. I think they are investing in the hospital. A cultural change is needed if we want primary care to function correctly and we want prevention and health promotion. It's a long term goal and more investment is needed. We only invest in the latest generation of scanners, and this, and that. Well that's well and good, because of course that's what people like [to see]. Having me prescribe quitting smoking or prescribe removing a certain food from a patient's diet … that doesn't have the same intrigue when it comes time to vote.

And another participant in center C5 described the need for prioritization of health promotion by higher management:

I think that the higher management is not conscious of the capacity for change that we in primary care can create for the population. I think they are not aware, because they are too near-sighted, focused on short-term processes. They are always making small changes, but they are not sufficient. I think that, if they were more conscious of the power of change that we [in primary care] have, they would give us more adequate tools. I think that, they likely know the theories because they are good managers with good advisors, I'm not saying otherwise. But you have to make the theory come to life in practice. If they were to drive theory-to-practice in primary care to provide an impetus for health promotion … self-care for populations according to age, according to pathology, taking into account the needs of the primary care centers, their personnel, the circumstances, the type of person they care for, that would be …. wow!

No facilitators were identified by participants in this area.

### III. Inner setting – E. Readiness for Implementation, 2. Available resources and D. Implementation climate, 2. Compatibility (i) with Workflow

These two constructs of inner setting were primarily, but not entirely, negatively assessed by the participants. Within compatibility with workflow and available resources, the participants predominantly reported of barriers. Specific barriers were related to available resources for implementation – primarily noting time constraints – and incompatibility with workflow, specifically describing multiple day-to-day demands to be managed, in addition to program implementation responsibilities.

One participant in center C7 commented about the insufficient consultation times to carry out the intervention:

When it's time for a patient's appointment, if I have time before he comes in, I have a look at the things we need to do/cover, like if I need to take his blood pressure or write up an order for blood work, and I look to see if he is missing PVS. I tried to integrate [PVS] into my consultations, but I do understand that it takes time, which is something I don't have. Sometimes I've contributed more, and sometimes less, depending on the time I have available and the priority given [to PVS]. If I have an appointment slot that lasts 35–40 [minutes], I try to finish [PVS] and move things forward. If I have time, I am able to involve myself in PVS and work on it [PVS].

One participant in center C1 explained the spillover effect of taking extra consultation time for the health promotion intervention:

You have 10 minutes per patient and in those 10 minutes you have address why he came, and that's it, not 1 minute more. And, of course, [extra time] is at the expenses of other patients. The minutes add up and [I decide] I have to stop for today, I can't dedicate any more time on this [PVS], I'm already so behind that it is hindering the rest [of my patients] today.

Another center C7 participant described how difficult health promotion was in practice:

Personally, I found it [PVS] very difficult to carry out. One appointment every 5 days at 20 minutes per patient, that 3 appointment slots, 4 slots, to specifically focus on this [PVS]. It seemed a little [difficult] …. to translate it to everyday practice.

Although facilitators were not commonly reported, some centers developed implementation strategies to address these barriers that facilitated integration of the health promotion intervention into daily or weekly primary care center routines. Numerous facilitators were identified by participants. For instance, some centers adapted the number of patients seen per day so that either (1) on one day per week, each professional had fewer patients with longer consultation times, or (2) they saw a few fewer patients each day to allow for one longer consultation timeslot. Other centers engaged in schedule sharing of patients or worked in mini-teams so that experienced professionals had extra time to carry out the health promotion program. On an ad hoc basis, some professionals carried out the PVS program with patients when they knew they had an open time slot after the scheduled appointment, thus permitting a longer consultation without getting behind.

### III. Inner setting – A. Structural characteristics, B. Networks and communications, C. Culture and D. Implementation climate, 6. Learning climate

These four CFIR inner setting constructs received a mixed evaluation by participants. Within learning climate, barriers were not commonly reported, whereas the participants reported both barriers and facilitators fairly equally in structural characteristics, networks and communications, and culture. Barriers within structural characteristics included being a large or recently formed center, where professionals did not know each other well, and having frequent changes in personnel. Barriers within network and communications included fragmented teams with poor communication. Physicians and nurses did not work together to cover their assigned patient population, and there was little communication between morning and afternoon shifts. When problems arose, informal communication routes such as coffee breaks were the only means to resolve them. The main barrier within culture and learning climate was a lack of unity/value of teamwork.

In an illustrative interaction of lack of communication, one center C7 physician asked another physician in the group:

Did your nurse partner participate [in PVS]? To which the other physician responded: No, not that I know of.

With regards to a culture of “every man for himself”, a center C5 participant describes the professionals at his/her center:

We weren't a very homogeneous group. Those of us who participated were like Pancho Villa's army, each one with a rifle.

The participants who positively evaluated these constructs offered useful facilitators for implementation of the health promotion intervention. Working in a small center where professionals and patients know each other was perceived to assist successful implementation. They valued having time set aside for weekly meetings with center professionals. These meetings were in place before the PVS program began and the centers often took advantage of these meetings to discuss progress implementing the health promotion intervention. Teamwork was described as a strong facilitator; the participants could ask on one another for support and advice, and a helping hand if needed. Furthermore, teamwork was perceived as valued by center leaders and colleagues.

One participant described the value of their small center C4:

Since we are [a small center] located in a small town, there is just one waiting room. The inhabitants know the health center professionals. I think that everyone knows about this [the PVS program] - exercise, diet. And [one specific nurse], even if she is not your assigned nurse, you know that she is the one who helps you quit smoking. The type of town, the health center layout, can be both good and bad.

A center C2 participant described the benefit of weekly meetings:

We'd meet weekly as a team. My team was an administrative assistant, the nurses, and two physicians. There [at the meeting] we would go over all the day-to-day issues that came up [over the past week]. We'd discuss problems we saw, for instance in the way appointments were scheduled [and resolving them]. We've been working less in these mini-teams, but that's how they worked, always dedicating some time to everyday questions and concerns.

One participant explained the culture of teamwork and good communication in place at center C4:

From an outsider's view, this primary care center works well as a team. In other centers where I've worked for many years, they haven't achieved this level of teamwork, far from it. From an outsider's standpoint, it's a good group of professionals, willing to involve themselves in their work [and PVS]. From an outsider's perspective, this was the most critical ingredient. When I started work here, they told me that everyone is participating and it's hard to say no when everyone else is doing it, working hard. And they communicate well, it's easy to obtain information and I think that is fundamental as well.

Another participant highlighted the supportive learning climate at center C1:

And I took a look at how others were using the PVS tool and wondered “how did they do that?”. So I asked my colleagues specific questions about everyday use of the tool [and they helped me]. I understood how it worked globally, but needed tips for day-to-day management, things I didn't know how to do. This [help] was fundamental.

These barriers and facilitators were also related to the construct III. Inner Setting–E. Implementation Climate, 3. Access to Knowledge and Information, which emerged in only two centers and was positively evaluated when it was mentioned. Only facilitators were reported. In addition to regular meetings to keep everyone informed, another facilitator was the availability of trainings to learn more about the PVS programs and information and communication technology to assist program implementation. The participants also indicated that they had the ability to attend these trainings (e.g. substitutions, flexibility in patient scheduling, etc.).

### III. Inner setting – D. Implementation climate (general)

The general implementation climate construct was also evaluated both negatively and positively by participants, with barriers predominately described. In general, participants perceived that an important factor for successful implementation was the percent of motivated professionals in a center participating in the PVS program. Two key barriers were individuals dropping out of the implementation of the program and less than a majority of professionals at a given center participating.

A center C4 participant described how each position at the center can play an important role in implementation:

I think the most important [factor] was to have everyone participating. If, starting from the administrative assistants, they can help patients fill out the survey [on baseline lifestyle], but then they go to see their physician or nurse who is not participating in the PVS program, then the effort was for nothing. In other words, I've filled out this survey, but my doctor or nurse is not saying anything about my responses …. When participation is selective, and not everyone is participating, it's much more complicated.

A motivating environment was noted as a facilitator in this general area of implementation climate.

A participant at center C4 notes how everyone, in every position was involved in implementation:

There were nurses who gave it [PVS] their all. The administrative assistants also tried to do everything they could, anything, doing the surveys, inputting the data. We committed [to the PVS program] and of course we tried to do everything we could.

### III. Inner setting – D. Implementation climate, 3. Relative priority

This construct received mixed evaluations by the participants with fairly equal numbers of barriers and facilitators reported. The specific barrier endorsed was having other competing programs introduced in the center at the same time as PVS was being implemented. The fact that PVS targeted systematic health promotion, which was valued by participants, was considered a facilitator that helped them give program implementation priority.

One participant described the extent to which he/she and her colleagues in center C2 prioritized the PVS program in response to the focus group facilitator's prompting.

What priority was PVS given at your center? We prioritized it, a lot! And with respect to other initiatives? We were obsessive about it. My patients even asked me if I had a fruit market or something. I told one older women that vegetables are very good for her and she did a good job eating vegetables, but she needed to eat a little more fruit. And she asked if I had a fruit stand in town. I just replied that no, that you just could use a little more fruit in your diet …. you have to find a way to reach each patient and that's one way, you know …

### III. Inner setting – E. Readiness for Implementation, 1. Leadership engagement

This construct was primarily, but not entirely, evaluated positively by participants. Facilitators were predominately reported. The key facilitator identified was having the PCP or nurse leaders at the center motivating staff to continuously participate in the program. The key barrier was the lack of this support.

One participant described the role of the PCP leader in ensuring a large majority of professionals in center C1 engaged in the PVS program.

Here we have a boss [PCP leader] who brings us all along with him/her. At the beginning, we were not very convinced, and already involved in other initiatives or with other work. But, at the end, we all ended up being involved. I didn't sign up for PVS at the beginning, but then he/she convinced me.

In contrast, a participant noted how the nurse leader did not contribute to such enthusiasm for the PVS program, leading to low nurse participation at center C7.

The nurse leader decided not to participate [in PVS] from the very beginning. I spoke with her about it two times. If she had said yes, the other nurses might have still said no, but not as forcefully. Come on, if we're all in this together, we can do it. But what happened was that the nurse leader said no, and then 3 or 4 are talking over coffee, and there you have it. That's why the nurses in general didn't participate.

These barriers and facilitators were related to those identified under the construct V. Process – B. Engaging, 2. Formally Appointed Internal Implementation Leaders, which emerged in only two centers and in which participants predominately reported facilitators.

### III. Inner setting – D. Implementation climate, 4. Organizational incentives and rewards

This construct was primarily, but not entirely, evaluated positively participants. Participants primarily reported facilitators. The facilitators mainly focused on ensuring that PVS participation was not a “punishment”. For instance, to address the barrier of the extra work involved in the PVS program, in some centers, the PCP leader was able to allow the professionals involved to recuperate some of the extra hours dedicated to work on the program, which facilitated implementation. Furthermore, the participants appreciated feeling valued for their participation in the program.

### Other constructs

Barriers and facilitators related to the constructs III. Inner Setting – D. Implementation Climate, 5. Goals and Feedback or III. Inner Setting – E. Readiness for Implementation (General) did not emerge in the analysis from any center.

### Table of most commonly reported barriers with facilitators

Table 3 summarizes the six main health care organization, management and policy barrier-facilitator pairs that emerged from the focus groups. These factors influenced the sustained implementation of an evidence-based health promotion program in primary care. To create this table, the barriers and facilitators were mapped so that the facilitators identified by participants from some centers addressed the barriers identified by participants in others. See Table 3 .

The majority of these barrier-facilitator pairs were identified from within the same construct. There were two exceptions: (1) The barrier of lack of financial and political support for health promotion implementation was reported by participants in all centers, and no facilitators within this construct were identified. Similarly, compatibility of health promotion with values of professionals working in primary care was reported by participants in all centers as an important facilitator and helped to overcome this barrier to program implementation facing all centers. Therefore, this barrier-facilitator pair was created. (2) The barrier of the lack of participation of a majority of professionals in a given center was important in the construct of implementation climate (general), but specific facilitating strategies did not emerge. However, the facilitators within organizational incentives and rewards, a sub-construct under implementation climate, were deemed to adequate address this barrier and this barrier-facilitator pair was created.

## Discussion

This study examined the perceptions of professionals in primary care centers in Basque Country, Spain working to integrate a health promotion intervention into their day-to-day routines. The results provide evidence for perceived health care organization, management, and policy barriers, and related facilitators, that influence successful implementation of health promotion in primary care. The barriers primarily related to lack of financial and political support, consultation time constraints, difficulty managing competing day-to-day demands, a lack of unity among center professional, poor communication among professionals and lack of majority of professionals in a given center participating. The main facilitators included a perception by professionals of health promotion as a valuable part of the health system and fundamental to primary care, rewarding (or not punishing) participation in the program and organizational and cultural changes focused on teamwork, schedule-sharing and flexible scheduling.

Compatibility with values of professionals working in primary care was endorsed by professionals from all centers and found to facilitate program implementation. There were no barriers in this regard. And this was related to tension for change to align day-to-day activities with these values. A systematic review using a meta-ethnography approach confirms the importance of this construct ( Rubio-Valera *et al.* , 2014 ); however, a scoping review suggests that only some primary health care professionals view health promotion as an integral part of their practice ( Peckham *et al.* , 2017 ). The phase II PVS pilot trial found tension for change to be associated with successful implementation ( Martinez *et al.* , 2017 ), and tension for change was found to be important to the successful implementation of a weight management program MOVE carried out in primary care centers of the US Veteran's Health Administration ( Damschroder and Lowery, 2013 ). It is possible that “Tension for Change” in those studies included quotations coded as compatibility with values in the present study. This new construct was distinguished from compatibility with workflow because the PVS program had been in development in the Basque Health System for a number of years. In other words, the program was not entirely new to participants, who were aware that change in other centers with respect to integration of health promotion in primary care had indeed occurred in prior phases of the PVS program and thus “Tension for Change” was less readily apparent.

External policy and incentives were evaluated as barriers to implementation by participants in all centers. No facilitators under this construct were identified. This construct also emerged from focus group discussions of PVS program implementation with professionals of the phase II pilot trial ( Martinez *et al.* , 2017 ). In that study, as in this one, all participants identified barriers to implementation in this construct. Because the evaluation was the same across centers, this construct was not associated with implementation effectiveness. In other words, it was a barrier in all centers, regardless of how well they were able to implement the intervention. In this study, it is a very important barrier that was frequently mentioned by participants. It could have an additive effect on implementation across all centers if adequately addressed. Future research could examine the effectiveness of implementation strategies designed to address this barrier. The CFIR-ERIC matching tool (see https://cfirguide.org/choosing-strategies/ ), for instance, suggests that 41% of experts identified two ERIC implementation strategies as potentially valuable: (1) altering incentives and/or allowance structures and (2) involving executive boards. Thirty-three percent of experts endorsed building a coalition as a further potentially effective implementation strategy ( Waltz *et al.* , 2019 ). As found in prior research ( Crable *et al.* , 2020 ) and in the present study, inner setting organizational incentives and rewards were primarily identified as facilitators, and it is possible that they may counteract outer setting barriers related to external policy and disincentives. Further analysis of the larger database of CFIR constructs examining the relationship with implementation success in this dataset is forthcoming.

Almost all quotations in compatibility with workflow were identified as barriers and focused on difficulties managing competing day-to-day demands in primary care while implementing the health promotion program. Within available resources, almost all of the quotations were barriers and primarily related to lack of time. Other studies support the importance of addressing barriers related to available resources. For instance, Crable *et al.* (2020) identified the specific barrier of staffing constraints in the implementation of improvement initiatives in safety net hospitals, which is directly related to the ability to manage patient demand while implementing the PVS program in primary care. Participants in this study indicated that they did not have anyone to cover their patients when they dedicated more time to the consultation to address health promotion and/or attend program-specific training. Prior research confirms this important barrier ( Alageel *et al.* , 2018 ; Brotons *et al.* , 2005 ; Damschroder *et al.* , 2011 ; Elwell *et al.* , 2013 ; Rubio-Valera *et al.* , 2014 ). This construct emerged from focus group discussions of PVS program implementation with participants of the phase II pilot trial ( Martinez *et al.* , 2017 ). It was not associated with successful implementation in that study because all participants from all centers identified lack of available resources as a barrier. If the levels do not differ across centers using the CFIR methodology, it is not related to implementation success. Yet, again, this construct should not be ignored in the implementation of a health promotion intervention in primary care. The fact that participants from all centers acknowledged its importance emphasizes how much it may be hindering implementation efforts. Further research into implementation strategies that might address these barriers is warranted.

Proportionally more facilitators than barriers emerged in the constructs of structural characteristics, network and communications, culture, learning climate and access to knowledge and resources. Other studies support these findings. The phase II PVS pilot trial found learning climate to be associated with successful implementation ( Martinez *et al.* , 2017 ) and also important to the successful implementation of the weight management program MOVE carried out in primary care centers of the US Veteran's Health Administration ( Damschroder and Lowery, 2013 ). More recently, the importance of strong communication as related to access to knowledge and information was identified as a facilitator of various improvement initiatives in safety net hospitals ( Crable *et al.* , 2020 ). In the phase II pilot trial of PVS, structural characteristics emerged but was not associated with implementation success across centers ( Martinez *et al.* , 2017 ). In that earlier study, all centers, regardless of implementation performance, evaluated this construct negatively. In this study, the evaluations were mixed, and it is possible that future analysis of implementation effectiveness with these data could show a relationship with successful implementation. Given that structural characteristics are more difficult to change, emphasis on implementation strategies to address or enhance factors related to networks and communication, culture and learning climate are recommended.

Regarding, implementation climate in general, a large proportion of quotations were identified as barriers to successful implementation. This construct was taken into account in the planning of the phase III PVS program trial evaluated in the present study. In order for a center to participate in the intervention arm, willingness to change was evaluated via the OR4KT ( Grandes *et al.* , 2017a ), and a majority of professionals at a given center had to commit to carrying it out. Even though this potential barrier was identified *a priori* , the results of the focus groups suggest that it was not sufficiently addressed as participants still evaluated the general implementation climate in their centers as negative. Some centers reached the 50% threshold for participation, but did not have participation from one specific group (such as nurses). Other centers had professionals dropout or move to a new center during the implementation phase. In the future, it may be appropriate to require that participation in PVS program implementation have higher thresholds of support from all types of center personnel – physicians, nurses and administrative assistants. The construct of leadership engagement, in which a large proportion of the quotations were positively evaluated by professionals, is considered to be an important facilitator in this regard.

## Implications and conclusions

The barriers and facilitators identified in this study represent contextual factors that are actionable by health care system managers and policy makers. In the present study, the CFIR domains and constructs were found to be valuable for program evaluation to structure the focus group discussion guide and analyze the resulting data. The CFIR was used as a tool to assist in the systematic identification of barriers and facilitators of implementation of an evidence-based health promotion intervention into the primary care setting. Almost all of the sub-set of CFIR constructs related to health care organization, management and policy were identified by professionals as relevant to successful integrating health promotion into routine primary care practice. The most important facilitator, as determined by participants from all centers, was related to compatibility with values, while the most important barriers identified by almost all participants in all centers were related to external policies and incentives, available resources and compatibility with workflow. Ongoing research with the larger dataset will examine the relationship between all CFIR constructs and actual implementation performance to explain variability in successful implementation at the center level.

When embarking on an attempt to integrate health promotion into primary care, the specific barriers and facilitators identified here could be considered by leadership for relevance. In addition, stakeholders could be consulted during program planning and development, guided by the CFIR, for instance, to ensure that context-specific issues are effectively addressed. Recommendations from implementation science (e.g. Nilsen, 2015 ) include assessing these factors prior to implementation of a specific evidence-based intervention or innovation in order to facilitate routine use in clinical practice. Uptake can also be enhanced by monitoring these constructs on a regular basis throughout intervention implementation. Including these constructs as process indicators (see Fernandez *et al.* , 2018 for available quantitative assessments) can help any potential barriers not previously identified to be promptly addressed. Similarly, any facilitators identified can be strengthened or, if setting-specific, can be tailored to different settings involved in the implementation.

Overall, the results of this and many other studies demonstrate that successful implementation of evidence-based interventions depends on contextual factors. Many, but not all, of these factors are modifiable. In the context of health promotion interventions and innovations, health system managers and policy makers have considerable influence to foster propitious environments. The exact actions needed will be context-specific. In this aspect, implementation science methodologies, including designs, theories, models and evaluation frameworks, can assist in this process of developing and evaluating appropriate implementation strategies that help to overcome identified barriers and leverage existing facilitators.

## Figures and Tables

**Table 1:** CFIR domains and constructs associated with health care organization, management and policy

CFIR domain	CFIR constructs related to healthcare organization, management, and policy
II. Outer setting	D. External policies and incentives
III. Inner setting	A. Structural characteristics B. Networks and communications C. Culture D. Implementation climate 1. Tension for change 2. Compatibility i. with workflow ii. With values 3. Relative priority 4. Organizational incentives and rewards 5. Goals and feedback 6. Learning climate E. Readiness for implementation 1. Leadership engagement 2. Available resources 3. Access to knowledge and information
V. Implementation process	B. Engaging 2. Formally appointed Internal implementation leaders (e.g. PCP leader)

**Table 2:** Focus group participants by role by participating center

Primary care center	Number of PCPs and pediatricians	Number of primary care nurses and midwives	Number of administrative assistants	Participation of the PCP leader?	Total participants
C1	3	2	1	Yes	6
C2	2	2	1	No	5
C3	2	2	2	Yes	6
C4	4	2	2	Yes	8
C5	4	7	0	No	11
C6	3	1	1	Yes	5
C7	6	2	0	Yes	8
*Total*	*24*	*18*	*7*	N/A	*49*

**Table 3:** Mapped health care organization, management and policy barriers with facilitators for health promotion program implementation in primary care

Barriers	Facilitators
1. Lack of financial and political support for health promotion implementation	1. Perception of health promotion as a valuable part of the health system and fundamental to primary care
2. Consultation time constraints	2A. Adapting the patient schedule to allow longer consultation times, either one each day or all on one day a week 2B. Taking advantage of open appointment times in schedule to have a longer consultation
3. Difficulty managing competing day-to-day demands	3. Schedule-sharing of patients or working in mini-teams to cover all patient needs
4. Lack of unity among center professionals	4. Fostering a culture that values teamwork
5. Poor communication	5. Scheduling weekly check-in meetings
6. Lack of majority of professionals in a given center participating	6. Encouraging commitment by not “punishing” participation, for instance by allowing participants to recuperate extra work hours dedicated to the program
